# A Case Report of an Ultrasound-Guided Popliteal Sciatic Nerve Block: An Asset for Emergency Lower Limb Debridement in a High-Risk Patient

**DOI:** 10.7759/cureus.57752

**Published:** 2024-04-07

**Authors:** Bhagyashri Soor, Ipshita Garg

**Affiliations:** 1 Anaesthesiology, Dr. D. Y. Patil Medical College, Hospital and Research Centre, Dr. D. Y. Patil Vidyapeeth, Maharashtra, IND

**Keywords:** sepsis-associated encephalopathy, adductor canal nerve block, popliteal sciatic nerve block, moderate hyponatremia, hypoglycemia, necrotizing fascitis

## Abstract

Severe sepsis, a syndrome characterized by systemic inflammation and acute organ dysfunction in response to infection, is a major healthcare problem affecting all age groups throughout the world. Sepsis-associated encephalopathy (SAE) is a common but poorly understood neurological complication of sepsis. It is characterized by diffuse brain dysfunction secondary to infection elsewhere in the body without overt central nervous system (CNS) infection. Such cases commonly present for emergency surgical management with inadequate fasting hours, limited time for preparation, and preoperative optimization. Regional blocks become the savior in such cases where both general and central neuraxial anesthesia become perilous. Here, we present a 70-year-old male, with a case of necrotizing fascitis of the left lower limb with septic encephalopathy, with compromised cardiac or respiratory function and deranged laboratory investigations. The patient was admitted for emergency lower limb debridement, and ultrasound-guided left lower limb popliteal sciatic nerve block along with an adductor canal block was chosen as the plan of anesthesia management.

## Introduction

Patients requiring emergency debridement usually present with sepsis, necrotizing fascitis, multi-organ dysfunction, and comorbid conditions. Sepsis-associated encephalopathy (SAE) leads to diffuse cerebral dysfunction induced by the systemic response to the infection leading to acute impairment in the level of consciousness and disorientation [1]. Manifestation of SAE can range from delirium to deep coma. Such patients may serve as a particular challenge to anesthesiologists. General anesthesia can be fatal due to profound hypotension and myocardial depression during induction. These patients mostly required mechanical ventilation postoperatively for tachypnea and respiratory compensation [2].

With the development of new techniques, such as ultrasound and peripheral nerve stimulators, the scope of anesthesia has shifted from general anesthesia and central neuraxial blockade for isolated limb surgery to peripheral nerve blocks. Here, we are reporting a case of septic encephalopathy with left lower limb necrotizing fascitis posted for emergency debridement and ultrasound-guided (USG) popliteal sciatic nerve block and adductor canal block being used as an effective alternative to general or central neuraxial anesthesia. 

## Case presentation

A 70-year-old male presented to us with complaints of an ulcer over his left foot persisting for five days, accompanied by dry cough and cold for a month and anorexia for three to four days. During the preoperative evaluation, the patient was highly agitated and restless. He was disoriented and noncooperative, which made it difficult to elicit a proper history. The patient seemed to be dehydrated and had no signs of pallor, icterus, cyanosis, edema, clubbing, or lymphadenopathy. Vitally, the heart rate (HR) was 86 bpm, blood pressure (BP) was 100/60 mmhg, respiration rate (RR) was 22/min, and SpO_2_ was 95% on room air. Systemic examination revealed muffled heart sounds and bilateral decreased air entry. The patient was edentulous. Airway assessment showed a three-finger mouth opening, mallampatti grade 2, and adequate neck movement. Along with elevated counts, the patient was in persistent hypoglycemia with blood sugar levels as low as 65 mg/dl even though the patient was a known diabetic for the past six months. The patient also had moderate hyponatremia with a deranged renal function test and a deranged coagulation profile. The ulcer was eventually diagnosed as necrotizing fasciitis. The patient was started on an injection piperacillin and tazobactam 2.25 gm IV tds. The routine laboratory investigations are mentioned in Table 1.

**Table 1:** Relevant routine investigations WNL: within normal limits

Parameters	Patient values	Reference range
Hemoglobin	12.3 g/dL	13.2-16.6g/dL
Total leukocyte counts	17,400/µL	4000-10000/µL
Platelets	1.24/µL	150000-410000/µL
Urea	185 mg/dL	17-49 mg/dL
Creatinine	4.7 mg/dL	0.6-1.35 mg/dL
Prothrombin time	20.7 seconds	11-13.5 seconds
International normalized ratio	1.76	0.85-1.15
Liver function test	WNL	-
Serology	Non-reactive	-
Random blood sugar	65 mg/dl	70-140 mg/dL
Serum sodium	128 mmol/L	136-145 mmol/L
Serum potassium	4.9 mmol/L	3.50-5.10 mmol/L
Serum albumin	2.6 g/dL	3.5-5.2 g/dL

**Figure 1:**
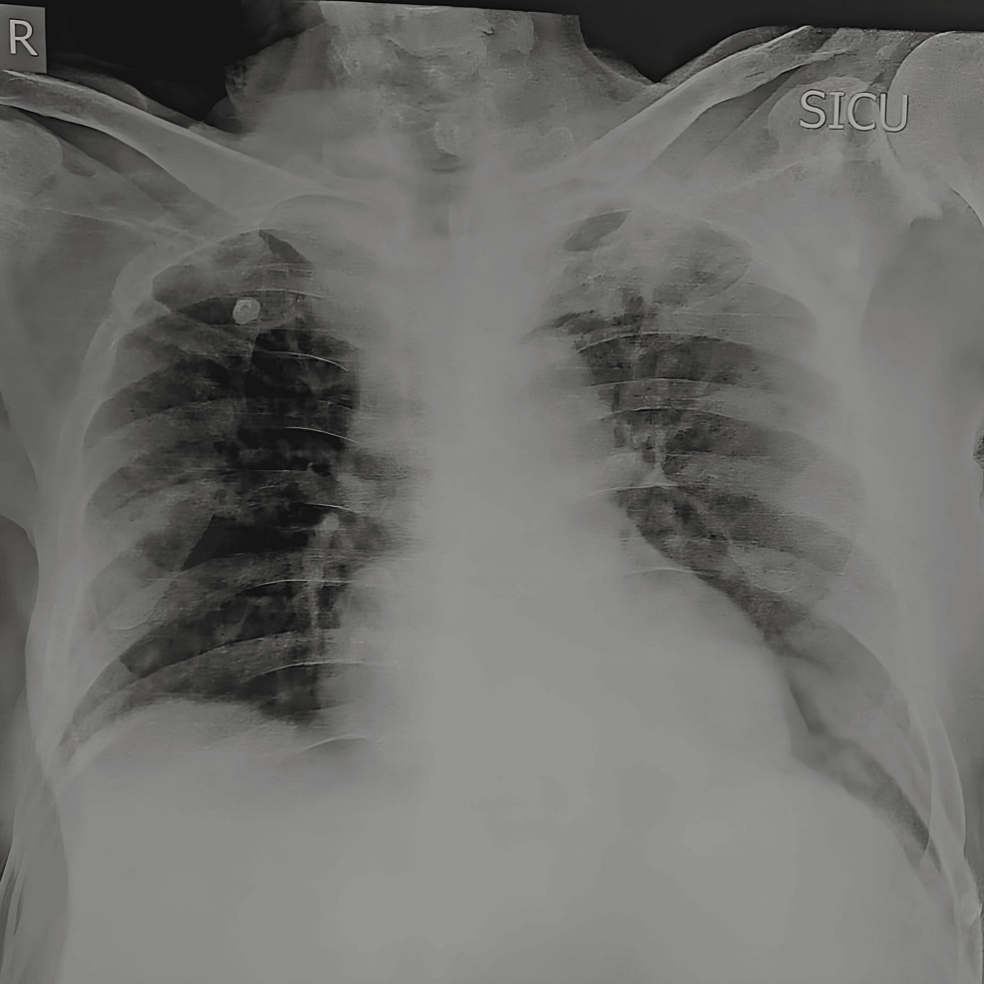
Chest X-ray on the day of admission showing the presence of ground-glass opacities in the left lung.

**Figure 2:**
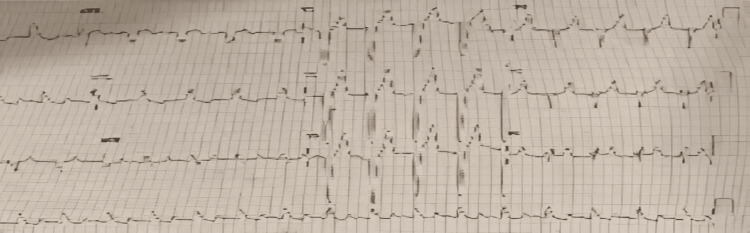
ECG on the day of admission with wide QRS complexes and features suggestive of a right bundle branch block.

The urine routine microscopy revealed that the urine was slightly turbid and that there was a presence of trace amount of proteins in it. The chest X-ray showed increased bronchovascular markings and the presence of ground-glass opacities in the left lung, as shown in Figure 1. ECG depicted wide QRS complexes and changes, suggestive of a right bundle branch block, as shown in Figure 2. Two-dimensional (2D) ECHO suggested ejection fraction of 25%, moderately depressed left ventricle (LV) function, dilated left atrium (LA), dilated LV, global LV hypokinesia, mild concentric LV hypertrophy, grade 1 diastolic dysfunction, aortic valve sclerosed, trivial aortic regurgitation, grade 3 mitral regurgitation, mild tricuspid regurgitation, mild pulmonary artery hypertension, and no clot/vegetation/effusion.

Intraoperative management

**Figure 3:**
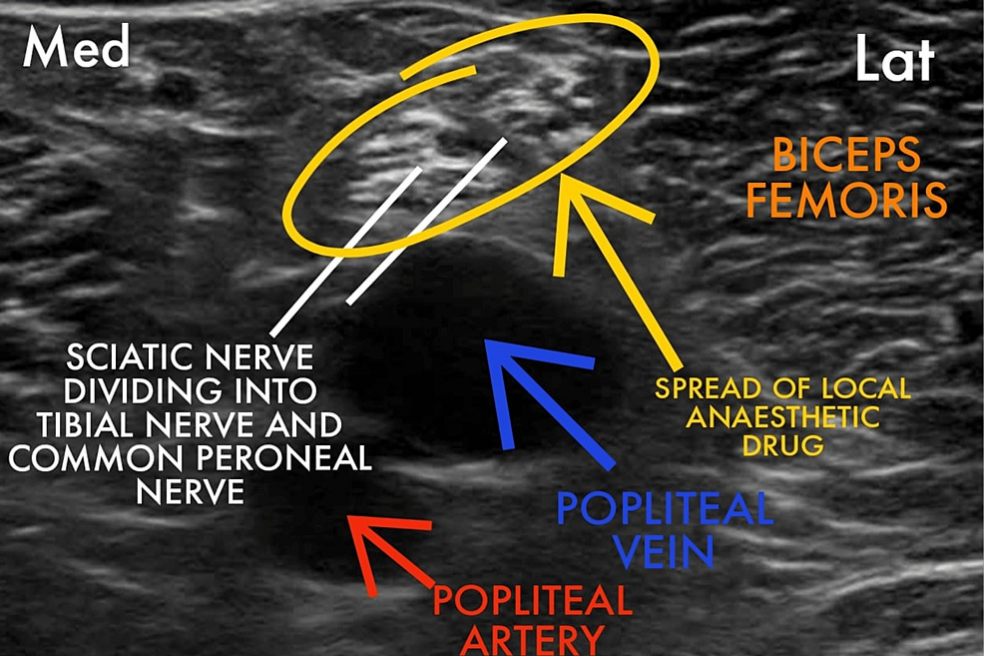
Ultrasound image showing the drug spread in the sciatic nerve sheath A needle was inserted from the lateral side to administer the block.

**Figure 4:**
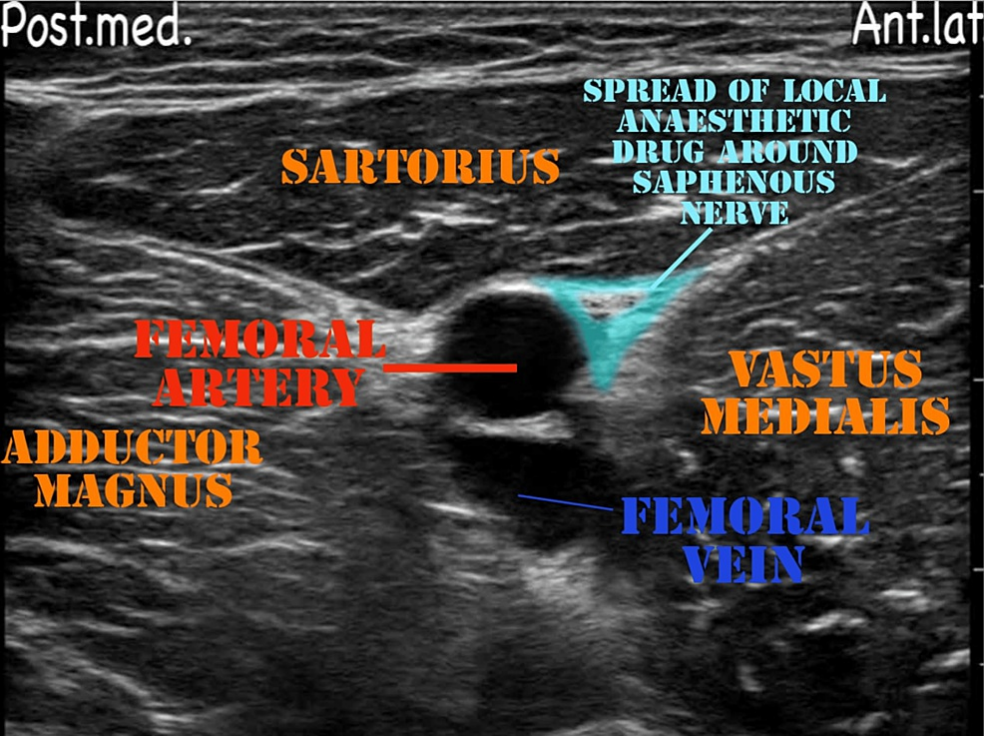
Adductor canal block Image showing the spread of the local anesthetic drug around the saphenous nerve.

The patient was assessed in the preoperative room. A wide bore intracath was secured in the right hand of the patient. Antibiotics and injection pantaprazole were administered intravenously half an hour prior to the incision time. The patient was taken inside the operation theater. Standard monitors were attached (electrocardiography, pulse oximetry, and non- invasive blood pressure) and 4l/min oxygen was delivered via a Hudson mask. D25 (in view of blood sugar level = 65 mg/dl) and injection paracetamol followed by normal saline (in view of hyponatremia) was given to optimize the blood sugar level (repeat bsl post D25 = 98 mg/dl) and hydration of the patient. The patient was given a right lateral decubitus position. An ultrasound machine (Philips Ultrasound Siemens ACUSON X300 Ultrasound Imaging System) was placed on the patient’s left side. The block was administered from the right side using a high-frequency linear array transducer probe and a 23-gauge spinal needle. A mixture of 25 cc of 0.5% ropivacaine with 8 mg dexamethasone was administered after calculating the toxic dose (considering the dose of ropivacaine for regional blocks as 3 mg/kg) as per the body weight of the patient. Under all aseptic precautions, a linear transducer was placed 3 cm above the popliteal fossa crease in a transverse orientation between the biceps femoris and semimembranosus and semitendinosus tendons. We inserted the needle in-plane of the probe and advanced the tip into the sciatic nerve sheath (Vloka’s sheath) between the tibial nerve and the common peroneal nerve. One to two ml of a local anesthetic was injected to confirm a proper needle tip position. A doughnut-shaped spread of the drug was visualized (Figure 3). After the block, we scanned 4-5 cm proximal to the injection site to confirm the spread of the drug within the sheath proximally. The mixture (15 cc) was administered. We also gave an adductor canal block, which covers the sensory supply of the medial side of the thigh. The patient's position was changed to supine. The thigh was abducted and externally rotated to give access to the medial 1/3rd of the thigh. The saphenous nerve was identified in the adductor canal, and 10 cc of the remaining drug was injected into the canal (Figure 4). Adequate spread of the drug was identified, and after eight to 10 minutes, an adequate sensory and motor blockade was achieved. Fluid administration was restricted in view of low ejection fraction, and no additional drugs were administered intravenously in view of known comorbidities. The intraoperative procedure was uneventful.

**Figure 5:**
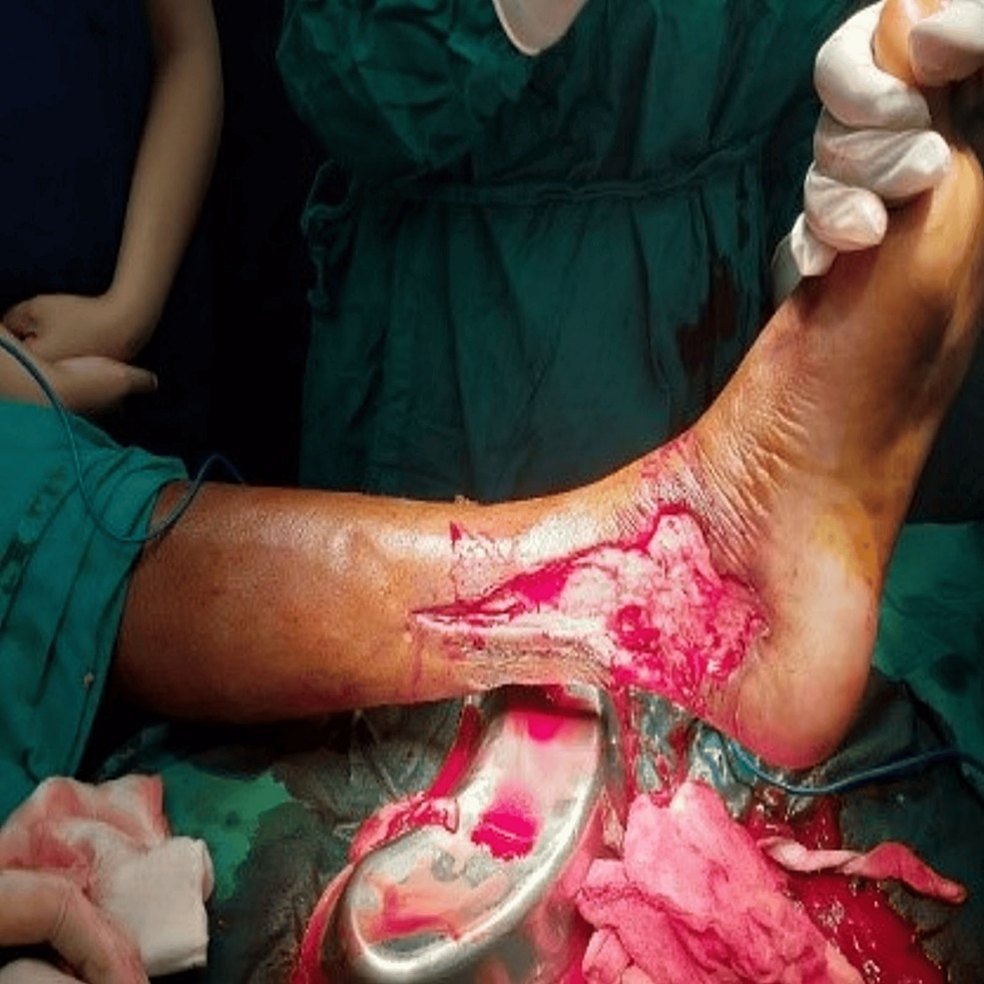
Septic area of the left foot

The septic foci (Figure 5) were debrided (intraoperative blood loss being around 250 ml), fasciotomy was done, and there was an immediate compartment pressure release. The patient was shifted to ICU for observation and further management. The postoperative counts came down to 11000/µL, and the pain at the surgical site was reduced.

## Discussion

Preoperative evaluation is of utmost importance. Pulmonary optimization and cardiac evaluation are to be taken care before posting the patient for surgery. Our patient presented to us in an emergency with no time to optimize the patient prior to the surgery, and from the surgeon’s perspective, the potential benefits of the surgery would have outweighed the risks. Managing such high-risk patients requires a reliable anesthetic technique to be chosen that will result in minimal hemodynamic changes.

Our patient was disoriented, delirious, agitated, and non-cooperative, indicating an SAE-like condition. Our targeted management included correcting hypoglycemia, and hyponatremia, avoiding fluid overload, and giving antibiotic cover (to manage sepsis). Neuraxial anesthesia can be catastrophic as it could lead to hypotension, bradycardia, meningitis, hematoma, post-dural puncture headache, neurological deficit, and more [3]. General anesthesia can result in high morbidity with significant hypotension, myocardial depression, and mechanical-ventilation-related complications.

**Figure 6:**
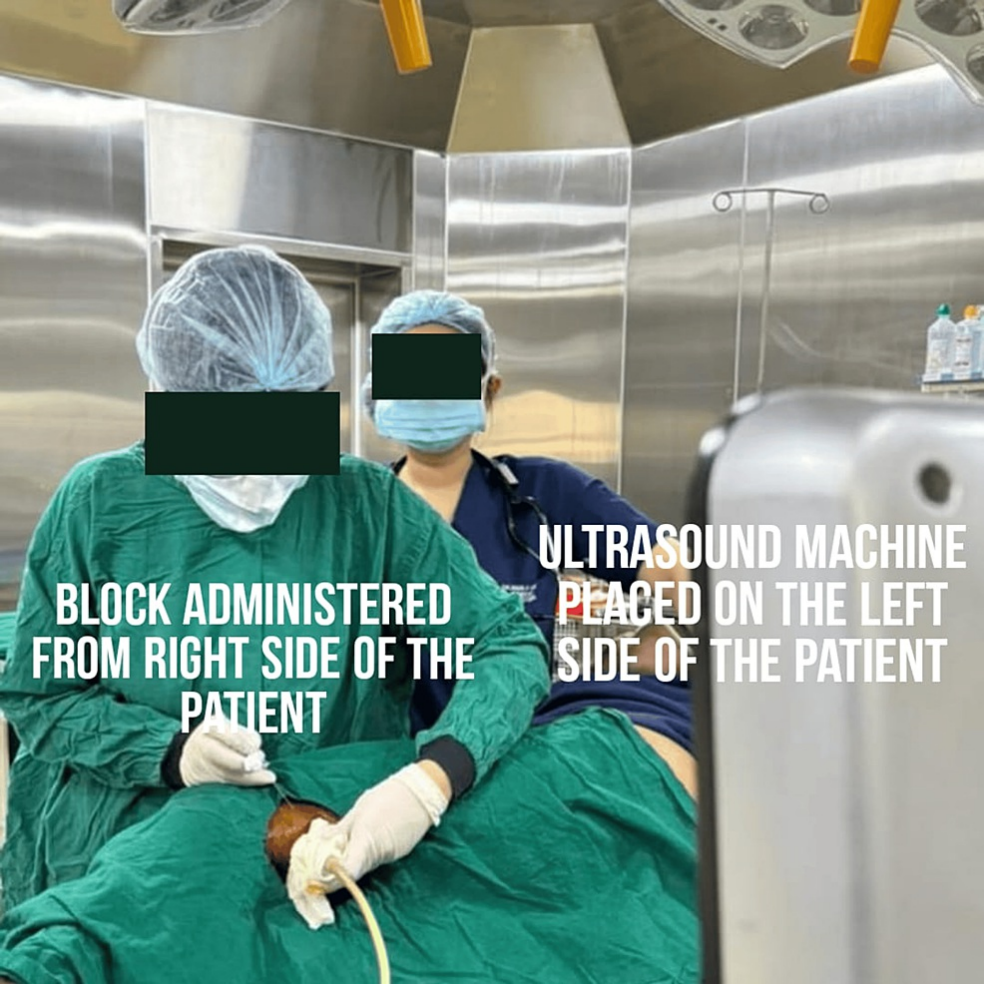
Ergonomics followed for administering the block The ultrasound machine was placed on the left side, and the block was administered from the right side of the patient. The administration of the block became convenient after following this ergonomics.

Ultrasound-guided peripheral nerve blocks remain a safe alternative approach. It is well tolerated with minimal cardiovascular instability and provides the advantages of pain relief intraoperatively and postoperatively with a prolonged block duration [3]. The ergonomics that we followed have shown ease in performing popliteal sciatic blocks with more probe stability, as shown in Figure 6. There is a real-time visualization of the distribution of local anesthetics, which helps in a faster onset and improves the quality and progression of sensorimotor blocks. It will avoid any blood vessel injury and will therefore prevent hematoma formation. Moreover, the use of ropivacaine improves the quality of blocks and provides prolonged duration of sensory blocks and postoperative analgesia. It provides more cardiac stability than bupivacaine [4]. Furthermore, calculated drug dosage will be delivered, which will decrease the incidence of local anaesthesia-related systemic toxicity and systemic complications related to general and spinal anesthesia [5]. A dual technique with a peripheral nerve stimulator and ultrasound guidance further increases the success rate and reduces the complications [6]. It ensures delivery of an adequate amount of drug, rapid block onset, prolonged duration pain relief, and decreased systemic complications. The onset of action of a peripheral nerve block is longer than a subarachnoid block, and the success rate for satisfactory blocks is lower than for neuraxial blocks. However, ultrasound-guided regional blocks make them worthwhile to perform especially in very sick patients [7,8].

## Conclusions

As discussed above, ultrasound guidance helped us visualize the anatomical structures, and we were able to deliver an adequate dose of the local anesthetic drug in the target area to achieve the desired motor and sensory block of the left lower limb. We were successfully able to avoid giving general or spinal anesthesia to the patient and hence the complications related to it. Therefore, ultrasound-guided PNB proved to be an excellent anesthetic technique in treating our high-risk patient with multiple comorbidities without compromising the patient's hemodynamics.
